# Insights into the Neutralization and DNA Binding of Toxin–Antitoxin System ParE_SO_-CopA_SO_ by Structure-Function Studies

**DOI:** 10.3390/microorganisms9122506

**Published:** 2021-12-03

**Authors:** Juan Zhou, Xue-Jian Du, Ying Liu, Zeng-Qiang Gao, Zhi Geng, Yu-Hui Dong, Heng Zhang

**Affiliations:** 1 Institute of Health Sciences and School of Life Science, Anhui University, Hefei 230601, China; zhoujuan@ihep.ac.cn (J.Z.); duxuejian@ihep.ac.cn (X.-J.D.); 2 Beijing Synchrotron Radiation Facility, Institute of High Energy Physics, Chinese Academy of Sciences, Beijing 100049, China; yingliu@ihep.ac.cn (Y.L.); gaozq@ihep.ac.cn (Z.-Q.G.); gengz@ihep.ac.cn (Z.G.); 3 University of Chinese Academy of Sciences, Beijing 100049, China

**Keywords:** toxin–antitoxin, ParE toxin, neutralization mechanism, crystal structure

## Abstract

ParE_SO_-CopA_SO_ is a new type II toxin–antitoxin (TA) system in prophage CP4So that plays an essential role in circular CP4So maintenance after the excision in *Shewanella oneidensis*. The toxin ParE_SO_ severely inhibits cell growth, while CopA_SO_ functions as an antitoxin to neutralize ParE_SO_ toxicity through direct interactions. However, the molecular mechanism of the neutralization and autoregulation of the TA operon transcription remains elusive. In this study, we determined the crystal structure of a ParE_SO_-CopA_SO_ complex that adopted an open V-shaped heterotetramer with the organization of ParE_SO_-(CopA_SO_)_2_-ParE_SO_. The structure showed that upon ParE_SO_ binding, the intrinsically disordered C-terminal domain of CopA_SO_ was induced to fold into a partially ordered conformation that bound into a positively charged and hydrophobic groove of ParE_SO_. Thermodynamics analysis showed the DNA-binding affinity of CopA_SO_ was remarkably higher than that of the purified TA complex, accompanied by the enthalpy change reversion from an exothermic reaction to an endothermic reaction. These results suggested ParE_SO_ acts as a de-repressor of the TA operon transcription at the toxin:antitoxin level of 1:1. Site-directed mutagenesis of ParE_SO_ identified His91 as the essential residue for its toxicity by cell toxicity assays. Our structure-function studies therefore elucidated the transcriptional regulation mechanism of the ParE_SO_-CopA_SO_ pair, and may help to understand the regulation of CP4So maintenance in *S. oneidensis*.

## 1. Introduction

Toxin–antitoxin (TA) loci are usually small genetic modules that are widespread in bacterial plasmids or chromosomes, and are typically composed of adjacent toxin and antitoxin genes encoding in operons. TA systems may regulate cell growth and death by targeting various cellular functions [1,2]. TA systems can be characterized into six different types (types I–VI) based on the interaction mode of the TA and the molecular nature of the antitoxin [3]. A group of type VII TA systems was recently discovered in several species [4], such as *Mycobacterium tuberculosis* [5] and *Shewanella oneidensis* [6], in which the antitoxins enzymatically neutralize toxins.

Type II TA is the most well-characterized and abundant system that is widely distributed in bacterial genomes and plasmids [3]. In type II TA systems, both the toxin and the antitoxin are proteins with small molecular weights, and the antitoxin can directly bind and neutralize the toxicity of the cognate toxin during normal growth by forming a tight protein–protein complex. The type II antitoxins are generally composed of a usually intrinsically disordered DNA-binding domain and a neighboring toxin-neutralizing domain [7]. The DNA-binding domain can bind to its own promoter region of the TA operon to autoregulate its transcription. The RelE/ParE superfamily is a prevalent toxin family that contains a conserved protein fold but a very low sequence conservation [8,9,10]. With the exception that *Escherichia coli* ParE2 did not inhibit DNA gyrase and *M. tuberculosis* ParE2 interacted weakly with GyrB [11,12], most ParE toxins are characterized as the inhibitors of bacterial gyrase by interacting with the GyrA subunit. They can inhibit gyrase-mediated supercoiling and therefore cause the accumulation of DNA breaks [13]. Distinctly from ParE toxins, the RelE subfamily toxins (such as RelE, YoeB, and HigB) function as ribosome-dependent ribonucleases [14,15,16]. They can cleave mRNA in a translation-dependent fashion to stall the ribosome [17,18].

The proteins SO_1444 (ParE_SO_) and SO_1445 (CopA_SO_) in the CP4_So_ prophage from *Shewanella oneidensis* were recently characterized as a typical type II TA pair, with an important role in CP4_So_ maintenance after its excision in host cells [19]. The toxin ParE_SO_ can severely inhibit cell growth and cause filamentous growth. The antitoxin CopA_SO_ can neutralize ParE_SO_ toxicity by direct and tight protein–protein binding. CopA_SO_ can bind to a DNA motif in the promoter region containing two inverted repeats (5′-GTANTAC (N)_3_GTANTAC-3′) to repress the transcription of the TA operon. CopA_SO_ can also bind to a highly similar DNA motif in the promoter region of another TA system, PemK_SO_/PemI_SO_, in megaplasmid pMR-1 of *S. oneidensis* to repress its transcription. The following NMR studies indicated CopA_SO_ has an ordered N-terminal domain and an intrinsically disordered C-terminal domain [20]. Crystal structure of the N-terminus showed it exhibits typical characteristics of ribbon–helix–helix (RHH) antitoxins for DNA binding. It was further speculated that the DNA binding of the N-terminal domain can be facilitated by the C-terminal domain, and such facilitation may further induce the fold and association of the C-terminal domain. The results indicated that DNA binding in the N-terminal domain will allosterically induce the disordered C-terminal domain to fold, which may further promote its self-association [20].

In spite of the above preliminary study on CopA_SO_, the inhibition mechanism of ParE_SO_ toxicity by CopA_SO_ and the autoregulation mechanism of the TA operon transcription remain uncharacterized. In this work, we performed the structure-function studies on ParE_SO_-CopA_SO_ system and demonstrated how the ParE_SO_ was recognized and inhibited by CopA_SO_. The promoter DNA-binding character of the antitoxin and essential residues for the toxicity of the toxin were also investigated. These findings revealed the neutralization mechanism of the ParE_SO_-CopA_SO_ TA pair, and helped to understand the autoregulation mechanism of this TA operon transcription.

## 2. Materials and Methods

### 2.1. Construction of Plasmids and Bacterial Strains

The recombinant plasmid pET28b expressing ParE_SO_-CopA_SO_ complex or CopA_SO_ was constructed as previously described [19]. The recombinant plasmid was transformed into an *E. coli* BL21(DE3) pLysS expression strain (Invitrogen, Waltham, MA, USA), for the expression and purification used in crystallization and DNA-binding studies. The gene encoding *copA_SO_-parE_SO_* or *parE_SO_* was cloned into pBAD18 by the method as described previously [21]. The pBAD18-based plasmids expressing ParE_SO_-CopA_SO_ or ParE_SO_ (wild-type and mutants) were transformed into *E. coli* K-12 strain TY0807 (*sup*^0^ *araD139 hsdR*Δ*lacX74 rpsL araD^+^*). These strains were grown in the presence of arabinose for cell toxicity assays. Site-directed mutagenesis of *parE_SO_* or *copA_SO_* was performed by a PCR-based technique according to the QuickChange site-directed mutagenesis strategy (Stratagene, San Diego, CA, USA) following the manufacturer’s instructions. The mutant genes were sequenced and found to contain only the desired mutations.

### 2.2. Protein Expression and Purification

Bacterial cells harboring pET28b-ParE_SO_-CopA_SO_ were grown to OD_600nm_ of 0.6 in LB media at 37 °C in the presence of 50 mg/mL kanamycin. Induction of protein expression was initiated by adding isopropyl-1-thio-β-D-galactopyranoside (IPTG) to the culture to a final concentration of 1 mM, and cells were grown at 16 °C. Cells were pelleted after 18 h by centrifugation at 3500 rpm for 35 min at 4 °C. Cell pellets were suspended in the buffer containing 20 mM Tris (pH 8.0), 250 mM NaCl, 10 µg/mL DNase, and 1 mM β-mercaptoethanol (β-ME). The cell suspension was disrupted by high-pressure homogenizer and then centrifuged at 16,000× *g* for 50 min at 4 °C. The supernatant was then loaded onto a Ni-NTA column that was pre-equilibrated with 20 mM Tris (pH 8.0), 500 mM NaCl, 10 mM imidazole, and 1 mM β-ME buffer. The His-tagged protein was eluted in 20 mM Tris (pH 8.0), 250 mM NaCl, and 250 mM imidazole. The complex was further purified by a Hitrap Q column (GE Healthcare, Chicago, IL, USA) pre-equilibrated with 20 mM Tris–HCl, 100 mM NaCl, 1 mM dithiotheitol (DTT), pH 8.0, with a linear gradient of 100–1000 mM NaCl in 20 mM Tris–HCl pH 8.0. Next, the protein was purified by Superdex-200 chromatography on an ÄKTA Prime system (GE Healthcare, USA) to obtain highly pure ParE_SO_-CopA_SO_ complex. The gel filtration buffer contained 20 mM Tris (pH 8.0), 100 mM NaCl, and 1 mM DTT. The eluted fractions in all purification steps were analyzed by sodium dodecyl sulfate–polyacrylamide gel electrophoresis (SDS-PAGE). CopA_SO_ was overexpressed and purified using the same procedures described above. 

The selenomethionine (Se-Met) ParE_SO_-CopA_SO_ complex was produced in minimal medium supplemented with 100 mg per liter lysine, phenylalanine, and threonine; and 50 mg per liter isoleucine, leucine, valine, and selenomethionine. The Se-Met protein production and purification were the same as described above. 

### 2.3. Protein Crystallization and Structure Determination

The purified complex was concentrated to ~10 mg/mL using a Millipore Amicon Ultra apparatus. The initial crystallization conditions were obtained through utilization of several sparse matrix screens (Hampton Research, Aliso Viejo, CA, USA) with the sitting drop vapor diffusion method at room temperature after 2–3 days. Crystal quality was optimized by adjusting the concentration of the precipitant and buffer. The best crystal of ParE_SO_-CopA_SO_ complex was obtained in a solution of 20% PEG3350 and 0.2 M tri-sodium citrate at 20 °C. The crystals were soaked in the reservoir solution supplemented with 15% (*v*/*v*) glycerol for a few seconds and then flash-frozen in liquid nitrogen. The diffraction data from a single crystal of a selenomethionine-substituted protein were collected on the beamline station BL19 U of the Shanghai Synchrotron Radiation Facility (SSRF) using+ a Pilatus 6M detector at a wavelength of 0.9788 Å. The total oscillation was 360° with 1° per image, and the exposure time was 1 s per image. 

All the data were processed by HKL2000 [22]. The Se-Met crystal structure of the ParE_SO_-CopA_SO_ complex was determined by the single-wavelength anomalous dispersion method. The selenium atoms were located by the program Shelxd and then used to calculate the initial phases in Shelxe [23]. The phases from Shelxe were improved in Resolve [24] and then used in Buccaneer [25] for model building. All structures above were refined with the program Phenix.refine [26] and manually corrected in Coot [27]. The qualities of the final models were validated with the program MolProbity [28]. Refinement statistics and model parameters are given in Table 1. The program PyMOL (http://www.pymol.sourceforge.net) was used to prepare structural figures.

### 2.4. Isothermal Titration Calorimetry (ITC)

ITC was applied to quantitatively determine the binding affinity of the ParE_SO_-CopA_SO_ complex or CopA_SO_ to a 25 bp DNA duplex derived from the *copA_SO_*-*parE_SO_* promoter (5′-TAAGGTATTACCTAGTAGTACTAAG-3′; the palindromic sequences are underlined). For the titration experiments, the protein was purified using the same method as above and was dialyzed against the buffer containing 20 mM Tris (pH 8.0), 100 mM NaCl, and 5% (*v*/*v*) glycerol for 24 h. DNA was dissolved in the same buffer as above. The ITC experiments were carried out using a high-sensitivity iTC-200 microcalorimeter from Microcal (GE Healthcare) at 20 °C using 100–400 μM DNA in the injector with 10–100 μM ParE_SO_-CopA_SO_ complex or CopA_SO_ in the sample cell. All samples were thoroughly degassed and then centrifuged to get rid of precipitates. Injection volumes of 2 µL per injection were used for the different experiments, and for every experiment, the heat of dilution for each ligand was measured and subtracted from the calorimetric titration experimental runs for the protein. Consecutive injections were separated by 2 min to allow the peak to return to the baseline. Integrated heat data obtained for the ITCs were fitted in a one-site model using a nonlinear least-squares minimization algorithm to a theoretical titration curve, using the MicroCal-Origin 7.0 software package.

### 2.5. Cell Toxicity Assay

*E. coli* K-12 cells harboring pBAD18-*copA_SO_-parE_SO_* (CopA_SO_ wild-type or mutants) or pBAD18-*parE_SO_* (ParE_SO_ wild-type or mutants) were grown in Luria–Bertani (LB) medium containing ampicillin (50 µg/mL) with or without 0.1 % L-arabinose. Optical densities at 660 nm (OD_660nm_) were measured every 2 h for individual cell cultures. Triplicate measurements were performed, and similar results were obtained for each measurement unless stated. The samples of the cells expressing His-tagged ParE_SO_ wild-type and variants were collected at 8 h, and their expression levels were examined by Western blot using anti-His antibodies. Each sample was lysed by ultrasonification to obtain soluble components after centrifugation. The total protein concentration was determined using the Bio-Rad protein-assay kit to confirm equal loading of lysate in each lane.

## 3. Results

### 3.1. ParE_SO_-CopA_SO_ Complex Has an Open V-shaped Structure

**Figure 1:**
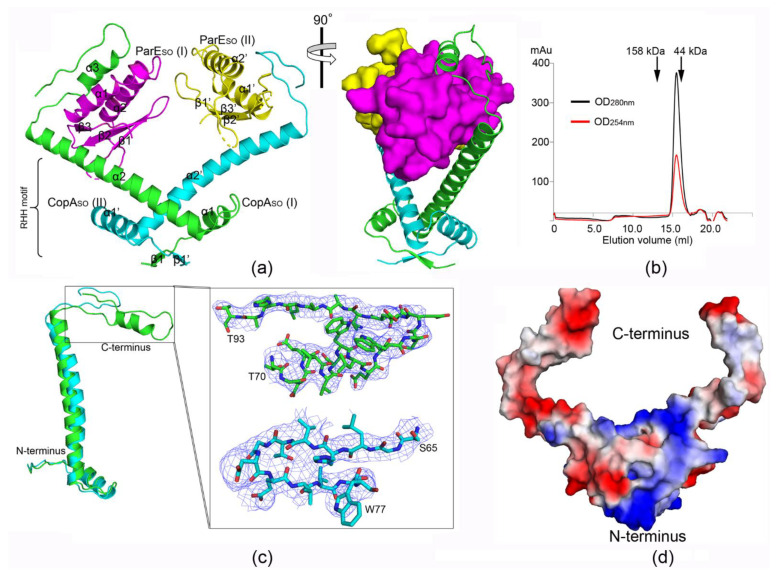
Overview of the crystal structure of the ParE_SO_-CopA_SO_ complex. (**a**) Overall structure of V-shaped ParE_SO_-(CopA_SO_)_2_-ParE_SO_ heterotetramer. The two CopA_SO_ molecules (I and II) are shown in cyan and green, respectively; whereas the two ParE_SO_ molecules (I and II) are shown in magenta and yellow, respectively. ParE_SO_ and CopA_SO_ are shown as illustrations, except that ParE_SO_ is shown as a surface in the right panel. (**b**) Estimation of the oligomeric state of the ParE_SO_-CopA_SO_ complex in solution using size-exclusion chromatography (Superdex^TM^ 200 10/300 GL). The purified complex was eluted from the chromatogram column at ~15.0 mL. (**c**) Structural superimposition of two CopA_SO_ molecules in this complex. An electron density map (2Fo-Fc) of the C-terminal regions of CopA_SO_ (I) (residues G66-T93) and CopA_SO_ (II) (residues G66-W77) is shown at a 2.0σ level. (**d**) The molecular surface representation of CopA_SO_ (blue, +6.9KT; red, −6.9KT), colored according to its local electrostatic potential. The N-terminal and C-terminal domains are distributed with predominantly positive and negative charges, respectively.

The crystal structure of the ParE_SO_-CopA_SO_ complex was determined by the single-wavelength anomalous dispersion (SAD) method using a Se-Met-labeled protein. The structure belonged to the space group *P4_3_2_1_2,* and was refined to a final *R*/*R*_free_ factor of 0.29/0.30 at 3.80 Å resolution (Table 1). There was a CopA_SO_ homodimer binding two respective ParE_SO_ molecules in an asymmetric unit (ASU). The resulting heterotetramer adopted an overall very open, V-shaped architecture with the organization of ParE_SO_-(CopA_SO_)_2_-ParE_SO_ (Figure 1a). The electron densities of the main chains in the overall structure, especially the C-terminus of CopA_SO_ (Figure 1c), could be well traced at moderate resolution. 

To determine the functional assembly of the ParE_SO_-CopA_SO_ complex, the oligomeric state of the purified complex in solution was estimated by gel filtration chromatography (Figure 1b). The complex migrated on size-exclusion chromatography with a similar elution volume to that of the marker protein (corresponding to a molecular mass of ~44 kDa). This was very close to the theoretical molecular weight of the heterotetramer observed in the crystal structure (~45.8 kDa).These results indicated the oligomeric state of the complex in solution was largely consistent with the heterotetramer crystal structure. 

### 3.2. CopA_SO_ Adopts a Partially Folded C-Terminal Conformation upon ParE_SO_ Binding 

The antitoxin CopA_SO_ is composed of an N-terminal RHH domain (β1, α1, and extended α2) and a C-terminal toxin-binding domain (α3 and the extended loop). The two neighboring CopA_SO_ molecules dimerize via the RHH domain to mediate the DNA binding. The characteristic intermolecular two-stranded antiparallel β-sheet (β1 and β1′) of the RHH motif is located in the dimer interface (Figure 1a). The C-terminus (helix α2) of CopA_SO_ extends outward from the dimerization domain, making the two ParE_SO_ toxins between two largely helical domains are sequestered (Figure 1a). Notably, the intrinsically disordered C-terminal domain is induced to fold into a partially ordered conformation upon ParE_SO_ binding. Moreover, the conformations of C-terminal extension in the two CopA_SO_ molecules are distinct, and part of the extension is further folded into a helix (α3) in CopA_SO_ (I) (Figure 1c). A surface-charge analysis of CopA_SO_ showed the N-terminal and C-terminal domains were distributed with dominantly positive and negative charges, respectively, which was consistent with the DNA-binding and toxin-binding functions, respectively (Figure 1d).

A DALI search (http://ekhidna.biocenter.helsinki.fi/dali_server) revealed the overall structure of CopA_SO_ did not show remarkable similarities with known structures, except the recently reported CopA_SO_ N-terminal structure (1–50 aa, PDB ID: 6IYA) (DALI Z-score of 5.1 and an RMSD of 1.2 Å). Structural comparison of the full-length CopA_SO_ with the N-terminal structure showed there were no remarkable changes in the RHH domain upon ParE_SO_ binding (Figure S1).

### 3.3. ParE_SO_ Is Structurally Homologous to ParE/RelE Toxins

**Figure 2:**
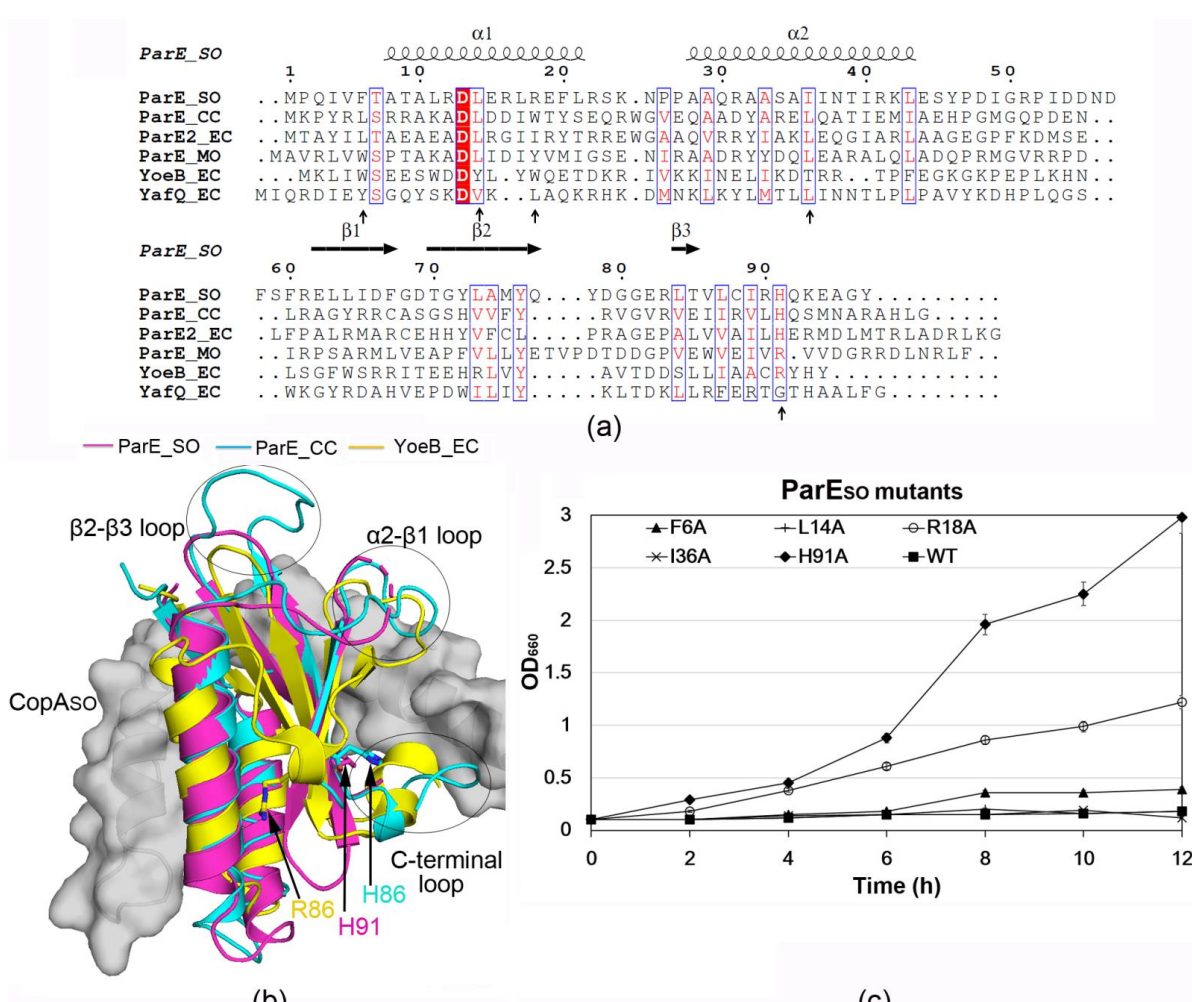
Identification of key residues involved ParE_SO_ toxicity. (**a**) Structure-based sequence alignment of ParE_SO_ with its representative homologs from different species performed using clustal X (version 1.81) and ESPript 3 (Robert and Gouet, 2014). The conserved residues are boxed in blue, identical conserved and low conserved residues are highlighted in red background and red letters, respectively. These homologs include ParE proteins from *Mesorhizobium opportunistum* (ParE _MO), *Caulobacter crescentus* (ParE_CC), and *Escherichia coli* O157 (ParE2_EC), as well as YoeB from *Escherichia coli* (YoeB_EC) and YafQ from *E. coli* (YafQ_EC). The CopA_SO_-binding residues mutated for the following cell toxicity assays are labeled using black arrows. (**b**) Structural superimposition of ParE_SO_ with ParE_CC (PDB ID: 3KXE) and YoeB_EC (PDB ID: 2A6Q). The major different regions are labeled using ellipses. The binding CopA_SO_ is shown as a gray surface. The critical residue His91 in ParE_SO_ and the corresponding residues His86/Arg86 in ParE_CC/YoeB_EC are shows as sticks. (**c**) Identification of key residues involved ParE_SO_ toxicity by cell toxicity assays. *E. coli* K-12 harboring pBAD18-*parE* (wild-type or mutants) were grown in LB medium containing 0.1% arabinose. Three independent experiments were conducted; error bars indicate standard error of mean (*n* = 3).

The toxin ParE_SO_ structure contains two N-terminal α-helices (α1-α2). The two helices form a hairpin tertiary structure that packs against the following three stranded antiparallel β-sheet (β1-β3) (Figure 1b and Figure 2a). The DALI search revealed that the overall structure of ParE_SO_ had remarkable similarities with several ParE homologs from different species (PDB IDs: 5CEG, 3KXE, 5CW7, et al.) [9,12,29], with DALI Z-scores of 10.4–11.4 and RMSDs of 1.4–2.3 Å, in spite of their low sequence identities (~20%). In addition, ParE_SO_ also had structural similarities with several RelE superfamily members, such as YoeB, YafQ, and HigB (PDB IDs: 2A6Q, 4MMG, 3BPQ, et al.) [30,31,32], with DALI Z-scores of 7.7–8.9 and RMSDs of 2.4–3.1 Å. In spite of the structural similarities of their overall structures, the conformations of several loops, including the α2-β1 loop, β2-β3 loop, and C-terminal loop, were distinct from each other (Figure 2b). In particular, the C-terminal loop of ParE_SO_ (His91) was directly involved in CopA_SO_ binding and played an important role associated with its toxicity (results below).

### 3.4. The C-Terminal Extension of CopA_SO_ Binds into a Positive and Hydrophobic Groove of ParE_SO_

**Figure 3:**
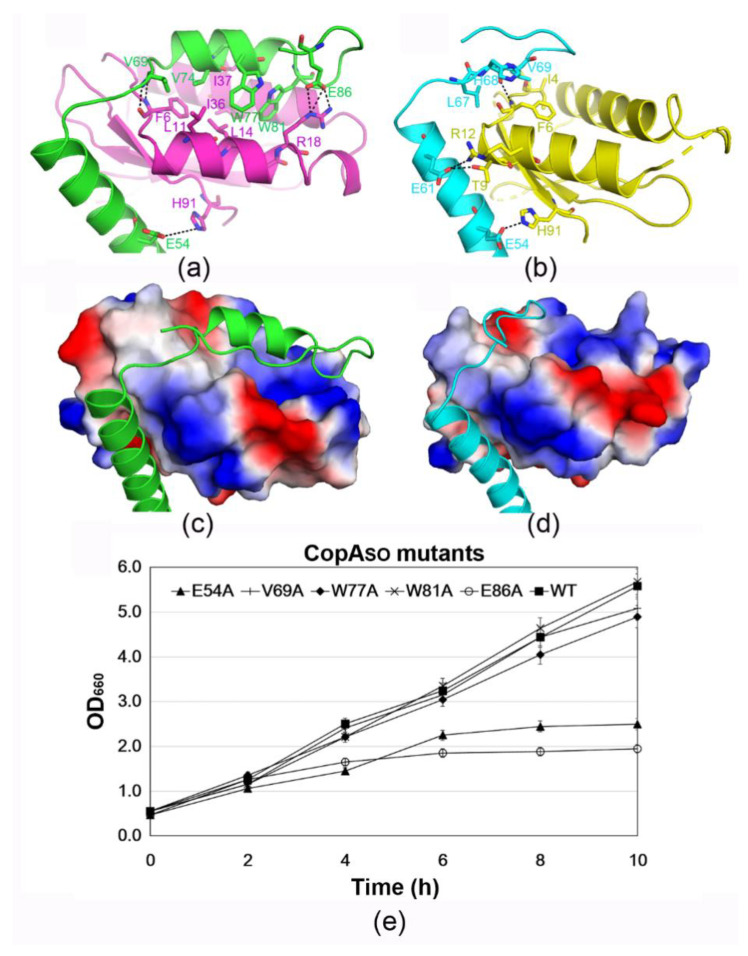
Contact analysis of ParE_SO_ and CopA_SO_ in the complex structure. (**a**,**b**) Detailed interaction analysis between ParE_SO_ and CopA_SO_. CopA_SO_ made extensive contacts, including H-bonds, salt bridges and hydrophobic interactions, with ParE_SO_. The distances within 3.8 Å are represented by the dashed lines. (**c**,**d**) The molecular surface representation of ParE_SO_ (blue, +6.3KT; red, −6.3KT), colored according to its local electrostatic potential. The C-terminus of CopA_SO_ were mainly distributed with negative charges (Figure 1d), which were well matched with the positive binding grove in ParE_SO_. (**e**) Inhibitory effect of CopA_SO_ (wild-type or mutants) on ParE_SO_ toxicity by determining *E. coli* growth. The CopA_SO_ mutants E86A abolished their inhibition on ParE_SO_-mediated cell toxicity and resulted in a reduction in cell growth. Three independent experiments were conducted; error bars indicate standard error of mean (*n* = 3).

Structural analysis showed that the toxin–antitoxin contacts were mainly mediated by the C-terminal domain (α3 and C-terminus of α2) of CopA_SO_ and the two helices (α1 and α2) of ParE_SO_ (Figure 3a,b). The buried surface area (1082 Å^2^) of CopA_SO_ (I) at the interface was significantly larger than that of CopA_SO_ (II) (580 Å^2^), which was 19.5%, and 10.3% of the total surface area (~5600 Å^2^), respectively. The loss of buried surface area was likely caused by the more disordered C-terminus of CopAso (II), in which only 77 residues could be modeled compared to the 93 residues in CopAso (I) (Figure 1c). Remarkably, surface-charge analysis of CopA_SO_ showed the C-terminal extension was predominantly distributed with negative charges (such as Glu54, Glu60, Glu61, and Glu86) (Figure 1d), which perfectly complemented positively charged regions in and around the binding grooves (such as Arg18, Arg22, and Arg90) (Figure 3c,d).

On the other hand, ParE_SO_ (I) and (II) were bound by the respective CopA_SO_ at a slightly different angle (~15°; Figure S2). Therefore, two ParE_SO_ molecules were not bound in a perfectly symmetric manner, as observed in common V-shaped TA structures such as ParD-ParE from *Caulobacter vibrioides* (PDB ID: 3KXE) and ParD3-ParE3 from *Mesorhizobium opportunistum* (PDB ID: 5CEG) [9,29]. The different binding angle of CopA_SO_ toward ParE_SO_ caused the specific contacts between the two ParE_SO_-CopA_SO_ pairs to be different. For example, the side chain of Glu86 in CopA_SO_ (I) can form three salt bridges with the side chain of Arg18. The main chain of Val69 in CopA_SO_ (I) can form two hydrogen bonds with the main chain of Phe6. The side chain of Glu61 in CopA_SO_ (II) can form a salt bridge and a hydrogen bond with the side chain of Thr9 and Arg12, respectively. Moreover, a hydrogen bond between the side chain of Glu54 and the side chain of His91 is formed in both CopA_SO_ molecules. Moreover, the binding groove of ParE_SO_ also mediates significant hydrophobic contacts with the C-terminal extension of CopA_SO_ in both ParE_SO_-CopA_SO_ pairs. In particular, the hydrophobic patches in ParE_SO_(I) are mainly composed of Phe6/Leu11/Leu14 in α1, and Ile36/Ile37 in α2. The two patches pack against the hydrophobic patch (Val69/Val74/Trp77/Trp81) of the C-terminal extension of CopA_SO_(I). The hydrophobic contacts in the other TA pair are mediated between Leu67/Val69 in CopA_SO_(II) and Ile4/Phe6 in ParE_SO_(II).

In order to identify the key residues of CopA_SO_ in toxicity inhibition, the interacting residues were mutated to alanine to test their neutralization capacity by cell toxicity assays (Figure 3e). The results showed that mutation of the interacting residues (Val69, Trp77, and Trp81) in CopA_SO_ could repress the toxicity to allow normal cell growth similar to the wild-type (Figure 3e). The mutants E54A and E86A had a notable effect on ParE_SO_ toxicity inhibition and caused a reduction in cell growth. Taken together, Glu54 and Glu86 were necessary for the inhibition of ParE_SO_-mediated cell toxicity. The critical roles of the two residues were consistent with their multiple salt bridges/hydrogen bonds with ParE_SO_ observed in the structure. Moreover, the solvation energy (Δ^i^G) of Glu54 and Glu86 is −0.19 and −0.43 kcal/mol, respectively, calculated from PISA analysis server (https://www.ebi.ac.uk/msd-srv/prot_int/pistart.html). Mutation of the two residues may greatly destabilize the interface and reduce the binding and inhibition of ParE_SO_ by CopA_SO_.

### 3.5. H91 Is a Key Residue for ParE_SO_ Toxicity 

To investigate whether the CopA_SO_-binding residues in ParE_SO_ are important for its cell toxicity, Phe6, Leu14, Arg18, Ile36, and His91 in ParE_SO_ were mutated to alanine. *E. coli* K-12 cells harboring pBAD18-*parE_SO_* (wild-type or mutants) were grown in the presence of arabinose (Figure 2c and Figure S3). The effects of these mutants on cell growth were studied. The mutants F6A, L14A, and I36A exhibited a similar growth reduction to that of the wild-type, indicating they did not affect the toxicity of ParE_SO_. The mutant H91A showed a significantly high growth rate, whereas R18A had a moderate effect on the cell growth. These results showed mutation of His91 caused a significant loss of the toxicity of ParE_SO_, and indicated that His91 was essential for the toxicity.

### 3.6. DNA-Binding Capacity of CopA_SO_ Is Inhibited upon ParE_SO_ Binding

**Figure 4:**
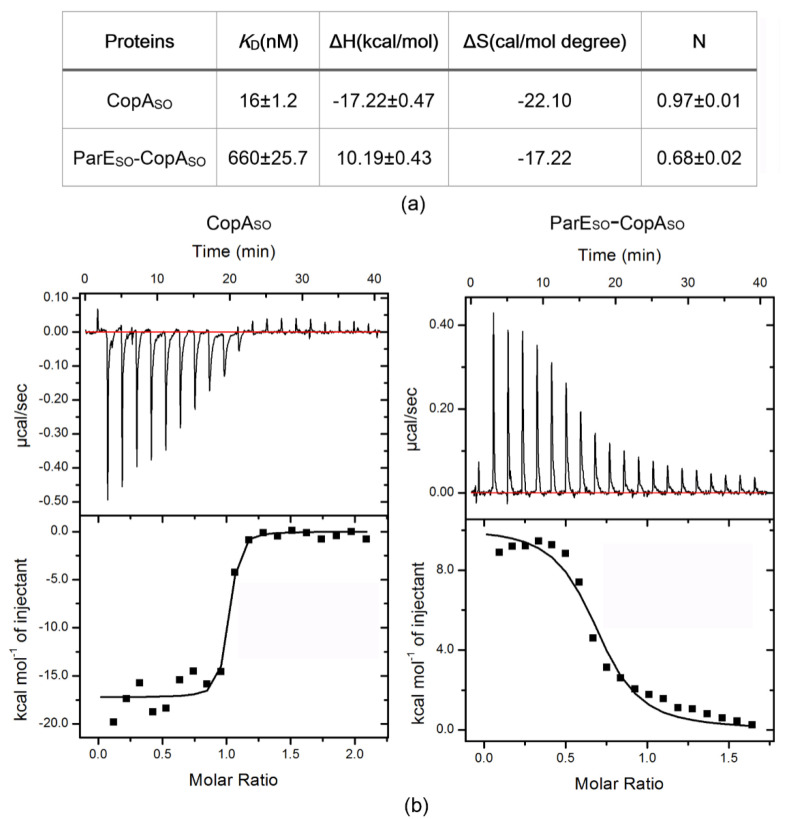
Thermodynamic analyses of CopA_SO_ and the ParE_SO_-CopA_SO_ complex binding to the promoter DNA by ITC. (**a**) ITC data for titration of the proteins with the DNA fragment. *K*_D_, binding affinity; ΔH, change in enthalpy; ΔS, change in entropy; N, number of binding site. (**b**) ITC spectra for CopA_SO_ and the ParEso-CopA_SO_ complex. Baseline subtracted raw ITC data for injections of DNA is indicated in the upper panels of each of the ITC profiles shown. The peaks normalized to the DNA/protein molar ratio were integrated as shown in the bottom panels. The solid dots indicate the experimental data, and the best fit to the experimental data was obtained from a nonlinear least squares method of fitting using a one-site binding model depicted by a solid line.

CopA_SO_ repressed the transcription of the TA operon by binding to a DNA motif in the promoter region containing two inverted repeats (5′-GTANTAC (N)_3_GTANTAC-3′) [19]. We generated a 25 bp DNA duplex corresponding to the promoter sequence containing the two inverted repeats, and explored the DNA-binding characteristics of CopA_SO_ by isothermal titration calorimetry (ITC) experiments (Figure 4). The results showed CopA_SO_ bound to the operator DNA fragment with a high affinity (*K_D_* = ~16 nM), which was significantly higher than that of the purified CopA_SO_-ParE_SO_ complex (*K_D_* = ~660 nM). More importantly, DNA binding of CopA_SO_ was an exothermic reaction (ΔH = −1.72 ×10^4^ M^−1^ cal/mol) that was converted to an endothermic reaction (ΔH = 1.02 ×10^4^ M^−1^ cal/mol) upon ParE_SO_ binding. The results suggested ParE_SO_ repressed the DNA-binding capacity of CopA_SO_ when two ParE_SO_ molecules bound to a CopA_SO_ dimer (TA ratio 1:1) as observed in the above structure. The reversion of the enthalpy change (ΔH) indicated that significant conformational changes of the RHH domain of CopA_SO_ may occur upon ParE_SO_ binding. Meanwhile, CopA_SO_ showed no detectable binding to the to the above 25 bp DNA fragment, in which the palindromic sequences were completely destroyed (Figure S4). This indicated the two inverted repeats were required for CopA_SO_-mediated transcriptional regulation.

## 4. Discussion

In this study, the structure of the ParE_SO_-CopA_SO_ complex revealed the TA pair is an open V-shaped heterotetramer. The intrinsically disordered C-terminal domain of CopA_SO_ was induced to fold into a partially ordered conformation upon ParE_SO_ binding. Structure-based mutagenesis on the interacting residues in ParE_SO_ and CopA_SO_ revealed the essential residues involved in the toxicity and the neutralization by cell toxicity assays, respectively. Our cell toxicity assays revealed Glu54 and Glu86 in CopA_SO_ are necessary for the inhibition of ParE_SO_ toxicity. Moreover, His91 and Arg18, which are the respective interacting residues of Glu54 and Glu86 in the complex structure, are important for ParE_SO_ toxicity. 

Structure analysis of ParE_SO_ showed that compared to RelE superfamily members, it is more structurally homologous to the ParE superfamily, in spite of their low sequence identities (~20%). Moreover, ParE_SO_ does not contain any of the three critical catalytic residues required for RNA cleavage on the ribosomes that are observed in the RelE family [17,18,30,31]. For example, the critical catalytic residues Glu46, Arg65, His83, and Tyr84, required for YoeB RNase activity [30], are not conserved in ParE_SO_ (corresponding to Gly50, Leu73, Lys93, and Glu94, respectively) (Figure 2a). Therefore, like most ParE superfamily members [11,12,33], ParE_SO_ may function as an inhibitor of DNA gyrase, and this hypothesis requires experimental validation. Because expression of ParE_SO_ has severe inhibition on *S. oneidensis* or *E. coli* growth, we were not able to obtain recombinant soluble ParE_SO_ by its overexpression or by purifying from the CopA_SO_-ParE_SO_ complex. A recent study on the antitoxin ParD2 structure from *Vibrio cholerae* (VcParD2) showed it had a similar organization to CopA_SO_, composed of the N-terminal ordered DNA-binding domain and the C-terminal intrinsically disordered protein (IDP) domain for ParE binding [34]. Moreover, in the absence of the IDP domain, VcParD2 was found to crystallize as a doughnut-shaped hexadecamer by the association of eight dimers. Analysis of the crystal packing of the N-terminal structure (1–50 aa, PDB ID: 6IYA) of CopA_SO_ or the TA complex structure in this study showed no such architecture could be observed like that in VcParD2. Moreover, both the full-length protein and the N-terminal domain of CopASO are likely dimers in solution. However, oligomerization of the full-length VcParD2 can generate a stable, open decamer or dodecamer in solution, likely resulting from entropic pressure from the IDP tails.

The autoregulation of TA operons transcription in some type II TAs, such as Phd/Doc, RelBE, and CcdAB, is controlled by a mechanism known as conditional cooperativity [7,35]. The operator DNA-binding affinities are controlled by different ratios of toxin to antitoxin in the cell, and therefore affect the transcriptional repression level of the TA locus. When the toxin:antitoxin level is below 1:1 (excess antitoxin), the toxin can function as a co-repressor to prevent the antitoxin:DNA interaction and affect transcription. On the contrary, at higher ratios than 1:1 (excess toxin), the toxin may bind and physically destabilize the preformed TA–DNA complex. The toxin therefore can act as a de-repressor and increase the toxin expression level by activating the TA operon [1,3]. For example, the transcriptional repression level of *relBE* operon is found to be regulated by the overall RelB:RelE ratio both in vivo and in vitro, and the antitoxin RelB alone binds the operator DNA with a relatively low affinity [35]. However, the binding is dramatically stimulated at a RelB:RelE ratio of 2:1, whereas the DNA binding is lost at the ratio of more than 2:1 by the conditional cooperativity mechanism [35,36]. Our ITC analysis showed free CopA_SO_ had a high binding affinity to the operator DNA (*K*_D_ = ~16 nM), whereas the binding was significantly inhibited (*K*_D_ = ~660 nM) when binding by ParE_SO_ at the 1:1 ratio. The results suggest that ParE_SO_ acted as a de-repressor rather than a co-repressor of the TA operon at the 1:1 ratio. On the contrary, at the T:A = 1:1 ratio, the DNA-binding capacity of HicA-HicB complex from *Streptococcus pneumoniae* was moderately higher than that of the antitoxin HicB dimer (*K*_D_ from ~8.8 μM to ~4.2 μM) [37]. This suggested that toxin HicA appeared to act as a co-repressor at the 1:1 ratio. Because we were not able to obtain recombinant ParE_SO_ under present conditions, the DNA bindings of CopA_SO_ in the presence of ParE_SO_ at different ratios were not determined. Further studies are required to determine whether the regulation of this TA operons transcription is controlled by conditional cooperativity. On the other hand, ParE_SO_ binding caused the enthalpy change (ΔH) reversion from an endothermic reaction to an exothermic reaction. The interaction changes due to hydrogen bond/salt bridge formation and van der Waals interactions may be reflected by binding enthalpy. The reversion of the enthalpy change indicated ParE_SO_ binding may induce significant conformational changes of RHH domains (such as the relative orientation) in the CopA_SO_ dimer, which in turn impacts the molecular basis for interaction with DNA. Recent NMR studies indicated the C-terminal domain of CopA_SO_ alone was intrinsically disordered [20]. Here, our structure showed ParE_SO_ binding induced the C-terminal domain of CopA_SO_ to fold and associate into a partially ordered conformation, including the extended α2 in both CopA_SO_ molecules and α3 in one molecule. 

In summary, our structure-function studies demonstrated the neutralization mechanism of the ParE_SO_-CopA_SO_ system and identified the key residues involved in ParE_SO_ toxicity. A previous study showed the repression of the mutated promoter (mutation of one or two inverted repeats) in the *parE_SO_*-*copA_SO_* operon by CopA_SO_ was greatly reduced [19]. Here, we found free CopA_SO_ had distinct DNA-binding thermodynamic characteristics from the ParE_SO_-CopA_SO_ complex. These biochemical studies are useful to understand the function of the ParE_SO_-CopA_SO_ TA pair associated with the stabilizing prophage CP4So in *S. oneidensis*.

## Figures and Tables

**Table 1:** X-ray data collection and refinement statistics.

Data Collection	ParE_SO_-CopA_SO_ Complex (Se-Met)
Beamline	SSRF 17U1
Wavelength (Å)	0.9788
Space group	*P*4_3_2_1_2
Unit-cell parameters	a = 85.4 Å, b = 85.4 Å, c = 110.9 Å, α = β = γ = 90°
Resolution (Å)	3.80 (3.87–3.80) ^a^
Number of unique reflections	7720 (375)
Completeness (%)	100 (99.7)
Redundancy	20.3 (22.8)
Mean I/ơ (I)	25.0 (7.2)
Molecules in asymmetric unit	4
R_merge_ (%)	19.4 (69.6)
R_meas_ (%)	19.7 (71.2)
CC_1/2_	100 (98.2)
**Structure Refinement**	
Reflections used in refinement	7667
Resolution range (Å)	33.93–3.80
R_work_/R_free_ (%)	29.8/30.5
Protein atoms	2618
Protein residues	312
Average B factor (Å^2^)	133.8
Ramachandran plot (%)	
Most favored	90.1
Allowed	6.6
Disallowed	3.6
R.m.s. deviations	
Bond lengths (Å)	0.005
Bond angles (°)	0.806

^a^ The values in parentheses are those for the highest resolution shell.

## Data Availability

The atomic coordinates and structure factors of the ParE_SO_-CopA_SO_ complex were deposited the RCSB Protein Data Bank with PDB code 7ETR.

## Supplementary Materials

The following are available online at https://www.mdpi.com/article/10.3390/microorganisms9122506/s1, Figure S1: Structural superimposition of CopA_SO_ derived from the complex with recently reported N-terminus (residues 1–49) of CopA_SO_. Figure S2: Structural superimposition of CopA_SO_ (I) and (II) showing that their corresponding ParE_SO_ molecules are bound at slightly different angles (~15°). Figure S3. Western blot of lysate from wild-type cells expressing His-tagged ParE_SO_ variants. Figure S4. CopA_SO_ binding to the mutated 25 bp DNA duplex (5′ TAAGGTATTACCTACCGGATGTAAG-3′) by ITC.
