# Supplementary material for: Insights into the Neutralization and DNA Binding of Toxin–Antitoxin System ParESO-CopASO by Structure-Function Studies
Source: Microorganisms. 2021 Dec 3;9(12):2506. doi: 10.3390/microorganisms9122506 (PMC8706911; doi:10.3390/microorganisms9122506)
Supplement: Supplementary file 1 [file microorganisms-09-02506-s001.zip › microorganisms-1382325-supplementary.pdf]

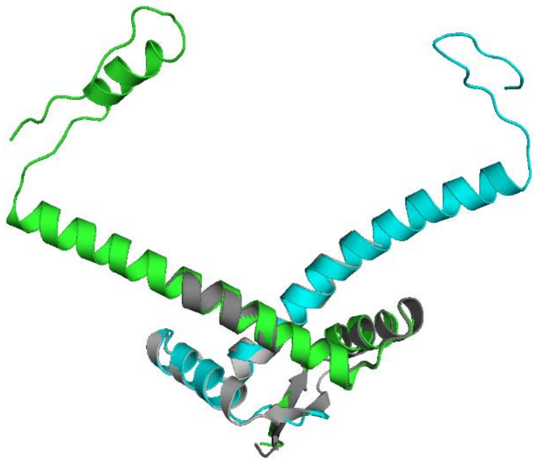

Figure S1. Structural superimposition of CopA$_{SO}$ derived from the complex (green and cyan) with recently reported N-terminus (residues 1-49) of CopA$_{SO}$ (PDB ID: 6IYA; light and heavy gray), revealing they have very similar structures.

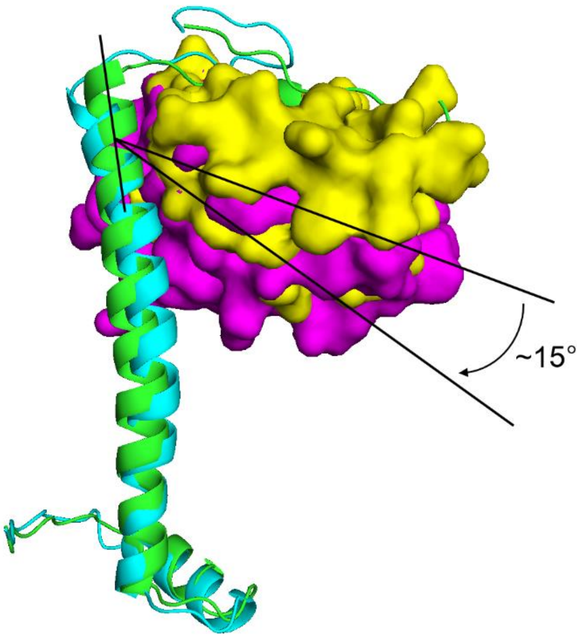

Figure S2. Structural superimposition of CopA$_{SO}$ (I) and (II) showing their corresponding ParE$_{SO}$ molecules are bound by slightly different angles (~15°).

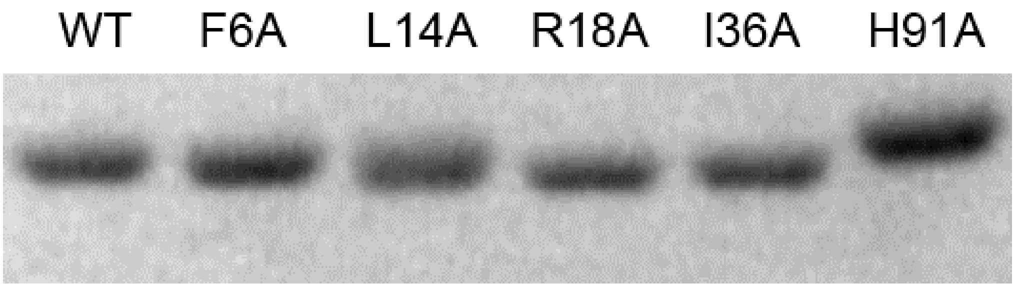

Figure S3. Western blot of lysate from wild-type cells expressing His-tagged ParE$_{SO}$ variants. The total protein concentration of each sample was determined using the Bio-Rad protein-assay kit to confirm equal loading of lysate in each lane.

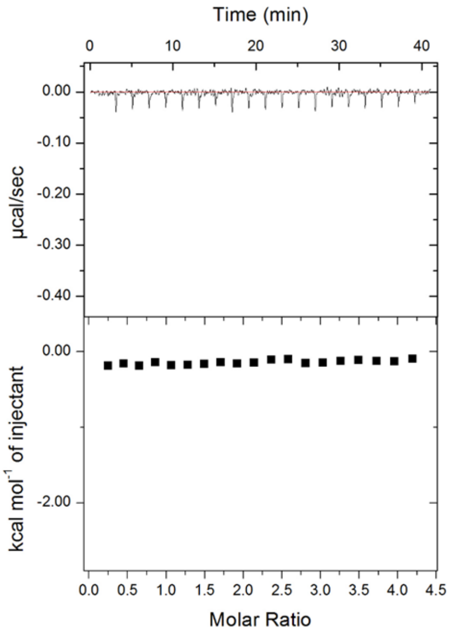

Figure S4. CopA_{SO} binding to the mutated 25-bp DNA duplex (5′ TAAGGTATTACCTA*CCG*G*ATG*TAAG-3′) by ITC. The underline sequences are two reverted repeats in the promoter region and the last one is mutated to the italic sequence.
